# Progression of myocardial fibrosis and functional clinical status in Hypertrophic Cardiomyopathy: a study with cardiac magnetic resonance

**DOI:** 10.1186/1532-429X-13-S1-P272

**Published:** 2011-02-02

**Authors:** Giancarlo Todiere, Giovanni Donato Aquaro, Alessandro Pingitore, Andrea Barison, Elisabetta Strata, Paola Capozza, Massimo Lombardi

**Affiliations:** 1 University of Pisa, Pisa, Italy; 2 Fondazione G.Monasterio MRI lab, Pisa, Italy; 3 IFC CNR, Pisa, Italy; 4 Scuola Superiore S.Anna, Pisa, Italy; 5 University of florence, florence, Italy; 6 Dipartimento Cardio Toracico, Pisa, Italy

## Introduction

Hypertrophic cardiomyopathy (HCM) is the most common genetic heart disease. Congestive symptoms of heart failure, such as effort dyspnea, may occur in this cardiovascular disorder. Myocardial fibrosis is a pathological feature of extracellular matrix remodeling, that may promote alterations in myocardial ventricular function. Magnetic Resonance Imaging (MRI) is now used as an non-invasive standard tool to detect focal myocardial fibrosis. The presence of scar using Delayed Enhancement technique (DE) is related to a worse prognosis in HCM. The relationship between the progression of myocardial fibrosis and the functional clinical status was not previously investigated.

## Purpose

a) to assess the severity and speed of progression of left ventricle (LV) fibrotic tissue formation by DE MRI b) to investigate if a relation between the rate of progression of LV scar and a worst clinical status exists.

## Methods

In 29 patients with HCM (20 males; mean age 42 ± 19 years) we repeated two cardiac magnetic resonance examinations, using a 1.5 Tesla scanner (GE Healthcare, Milwaukee, USA). We evaluated LV mass and volumes and LVEF by the acquisition of conventional SSFP cine short axis images. DE was detected using T1 GRE IR acquired 10 minutes following contrast injection. Quantification of DE was performed as previously described (1).

## Results

At the first CMR DE was detected in 21 subjects (72%), with an average extent of 8.1 ± 8 % of LV mass. CMR was repeated after an average of 772 ± 300 days. At the second CMR exam DE was found in 28 patients (97%). Among them, 16 subjects showed a significant increase of extent of DE (mean augment of 6.9 ± 5.5%, p< 0.0001). During the time interval between the 2 examination, NYHA class worsened in 10 patients who presented higher increase of DE extent than those with preserved functional status (8.3 ± 6.8 vs 3.4 ± 3.1, p < 0.04).

## Conclusions

From these data we conclude that the progression of fibrosis in HCM is fast. The increase of extent of fibrosis in these patients is related to a worse clinical status. Therefore MRI can be applied as an useful safe tool, for longitudinal follow up evaluation of progression of the disease.
